# Letter to the editor: Sequencing bias for residue 28 of the neuraminidase of the recent highly pathogenic avian influenza virus A(H5N8)

**DOI:** 10.2807/1560-7917.ES.2021.26.36.2100829

**Published:** 2021-09-09

**Authors:** Jinyue Guo, Congying Wang, Shujian Huang, Feng Wen

**Affiliations:** 1 College of Life Science and Engineering, Foshan University, Foshan, Guangdong, China

**Keywords:** highly pathogenic avian influenza virus, H5N8, NA, Sequencing

**To the editor:** We read with great interest the recent rapid communication by Pyankova et al. entitled ‘*Isolation of clade 2.3.4.4b A(H5N8), a highly pathogenic avian influenza virus, from a worker during an outbreak on a poultry farm, Russia, December 2020*’ [1]. This report described the genetic characterisation of the first human case of a highly pathogenic avian influenza virus (HPAIV) A(H5N8) isolated from a poultry worker in Russia. We would like to share our perspective on the polymorphism of residue 28 of the neuraminidase of influenza A(H5N8) viruses.

The re-emergence of HPAIV of subtype A(H5N8) globally is a concern for both the poultry industry and public health [2]. In December 2020, Pyankova et al. collected nasopharyngeal swabs from 37 poultry farm workers during an outbreak of A(H5N8) HPAIV in the Astrakhan region in southern Russia. Their whole genome sequencing results suggested that the human isolate, passaged on MDCK cells, had an amino acid substitution S28N in the neuraminidase (NA) protein, while all clinical samples had the 28S amino acid. As the authors themselves write “*The absence of 28N in NA sequences of clinical specimens could be due to bias that may have been introduced by using nested PCR*”. However, we would like to point out that the absence of 28N in NA sequences is not likely to be a sequencing bias by nested PCR.

Since November 2020, our diagnostic laboratory has performed surveillance for avian influenza virus in domestic geese in Guangdong, China. RNA was extracted directly from clinical samples with Body Fluid Viral DNA/RNA Miniprep kit (Axygen, Hangzhou, China) and the reverse transcription was performed with a Uni-12 primer. A total of 10 influenza A(H5N8) viruses were detected and the NA genes were obtained with a set of universal primers described by Hoffmann et al. [3]. Genes were subcloned into the pMD-18T vector and sequenced by Sanger sequencing. Sequencing results were deposited at GenBank with accession numbers MZ882169-MZ882176 (haemagglutinin (HA)) and MZ882177-MZ882184 (NA). Our newly identified influenza A(H5N8) strains from geese shared high nucleic acid homology with the A/Astrakhan/3212/2020 strain (99–99.8% for NA, 98.9–99.7% for HA). Of note, all 10 influenza A(H5N8) isolates had 28S in NA. Furthermore, a sequence comparison of the NA gene revealed that residue 28 had a low diversity with an entropy of 0.042. The substitution S28N was found in only 0.39% (7/1,789) of the influenza A(H5N8) strains worldwide (GISAID by 28 August 2021). All closely related influenza A(H5N8) viruses of clade 2.3.4.4b reported in GISAID since November 2020 had 28S in NA. Pyankova et al. claimed that the human strain (A/Astrakhan/3212/2020) and one (A/chicken/Astrakhan/321–06/2020) of five avian strains had 28N in NA, however, no original sequence was available for these two isolates.

Notwithstanding, we fully agree that the amino acid substitution S28N occurs only rarely and sporadically. We propose that the absence of 28N in NA sequences of clinical specimens is normal and is not likely to be a sequencing bias by nested PCR. The presence of 28N in the human isolate could be a result of cell adaptation during the passaging of the virus in MDCK cells.
